# Di­chlorido­[*N*-(*N*,*N*-di­methyl­carbamimido­yl)-*N*′,*N*′,4-tri­methyl­benzohydrazonamide]­platinum(II) nitro­methane hemisolvate

**DOI:** 10.1107/S1600536814003894

**Published:** 2014-03-12

**Authors:** Dmitrii S. Bolotin, Nadezha A. Bokach, Matti Haukka

**Affiliations:** a Department of Chemistry, Saint Petersburg State University, Universitetsky Pr. 26, 198504 Stary Petergof, Russian Federation; b Department of Chemistry, University of Jyväskylä, Finland, PO Box 35, FI-40014University of Jyväskylä, Finland

## Abstract

In the title compound, [PtCl_2_(C_13_H_21_N_5_)]·0.5CH_3_NO_2_, the Pt^II^ atom is coordinated in a slightly distorted square-planar geometry by two Cl atoms and two N atoms of the bidentate ligand. The (1,3,5-tri­aza­penta­diene)Pt^II^ metalla ring is slightly bent and does not conjugate with the aromatic ring. In the crystal, N—H⋯Cl hydrogen bonds link the complex mol­ecules, forming chains along [001]. The nitromethane solvent molecule shows half-occupancy and is disordered over two sets of sites about an inversion centre.

## Related literature   

For the luminescent properties of 1,3,5-tri­aza­penta­diene metal complexes, see: Gushchin *et al.* (2008 ▶); Kopylovich & Pombeiro (2011 ▶); Sarova *et al.* (2006 ▶) and for the catalytic activity of related complexes, see: Kopylovich & Pombeiro (2011 ▶). For the synthesis of [PtCl_2_(C_13_H_21_N_5_)] and similar compounds, see: Bolotin *et al.* (2013 ▶). For standard bond lengths, see: Allen *et al.* (1987 ▶); Orpen *et al.* (1989 ▶).
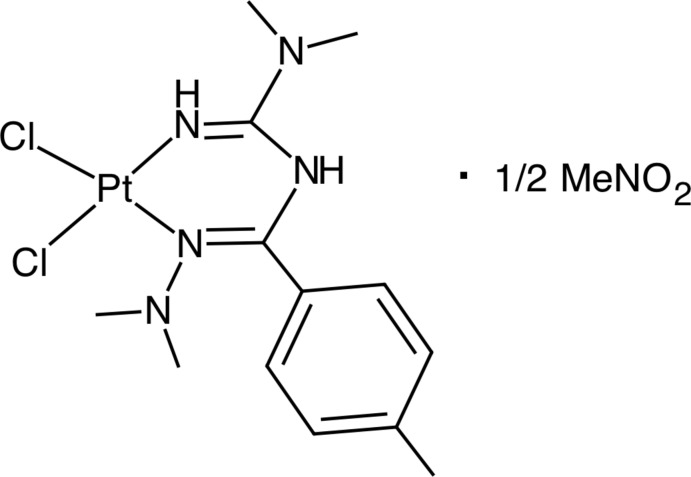


## Experimental   

### 

#### Crystal data  

[PtCl_2_(C_13_H_21_N_5_)]·0.5CH_3_NO_2_
*M* *_r_* = 543.86
Monoclinic, 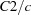

*a* = 20.7561 (3) Å
*b* = 15.1847 (3) Å
*c* = 14.5191 (2) Å
β = 126.255 (1)°
*V* = 3690.1 (1) Å^3^
*Z* = 8
Mo *K*α radiation
μ = 7.91 mm^−1^
*T* = 100 K
0.44 × 0.29 × 0.20 mm

#### Data collection  

Bruker Kappa APEXII DUO CCD diffractometer
Absorption correction: numerical (*SADABS*; Sheldrick, 2008*a* ▶) *T*_min_ = 0.130, *T*_max_ = 0.297
38976 measured reflections
9015 independent reflections
8276 reflections with *I* > 2σ(*I*)
*R*_int_ = 0.017

#### Refinement  

*R*[*F* ^2^ > 2σ(*F* ^2^)] = 0.015
*wR*(*F* ^2^) = 0.035
*S* = 1.06
9015 reflections
226 parameters
2 restraints
H-atom parameters constrained
Δρ_max_ = 0.78 e Å^−3^
Δρ_min_ = −1.01 e Å^−3^

### 

Data collection: *APEX2* (Bruker, 2010 ▶); cell refinement: *SAINT* (Bruker, 2010 ▶); data reduction: *SAINT*; program(s) used to solve structure: *SHELXS97* (Sheldrick, 2008*b* ▶); program(s) used to refine structure: *SHELXL97* (Sheldrick, 2008*b* ▶); molecular graphics: *CrystalMaker* (CrystalMaker Software, 2011 ▶); software used to prepare material for publication: *SHELXL97*.

## Supplementary Material

Crystal structure: contains datablock(s) I. DOI: 10.1107/S1600536814003894/zq2215sup1.cif

Structure factors: contains datablock(s) I. DOI: 10.1107/S1600536814003894/zq2215Isup2.hkl

CCDC reference: 987902

Additional supporting information:  crystallographic information; 3D view; checkCIF report

## Figures and Tables

**Table 1:** Selected bond lengths (Å)

Pt1—N5	1.9809 (11)
Pt1—N1	2.0309 (11)
Pt1—Cl1	2.3223 (3)
Pt1—Cl2	2.3279 (3)

**Table 2:** Hydrogen-bond geometry (Å, °)

*D*—H⋯*A*	*D*—H	H⋯*A*	*D*⋯*A*	*D*—H⋯*A*
N3—H3*N*⋯Cl1^i^	0.87	2.45	3.2154 (11)	147
N5—H5*N*⋯Cl1^ii^	0.84	2.67	3.4598 (12)	157

Symmetry codes: (i) 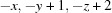; (ii) 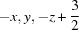.

**Fig. 1.**
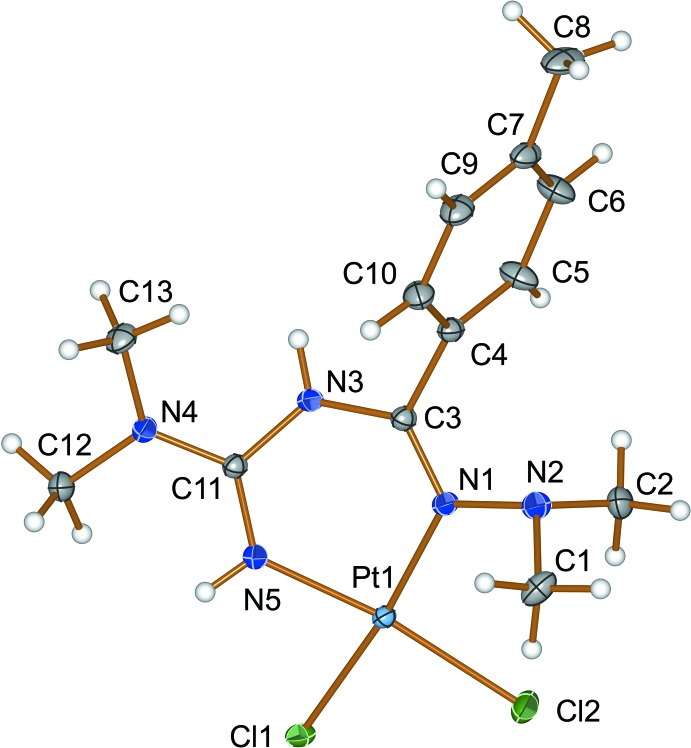
View of the title complex. Thermal ellipsoids are drawn at the 50% probability level. Pt atoms are pale blue, chlorine, carbon, and nitrogen atoms are green, grey, and blue, respectively. The disordered nitromethane of crystallization has been omitted for clarity.

## supplementary crystallographic information

### 1. Introduction

The title complex (I) was obtained in the framework of our project devoted to the intra­molecular rearrangement of carbamimidoyl­amidoxime and di­alkyl­cyanamide ligands to furnish amidrazone complexes (Bolotin *et al.*, 2013). The luminescent properties of 1,3,5-tri­aza­penta­diene metal complexes and the catalytic activity of some related complexes have been reported in the literature (Gushchin *et al.*, 2008; Kopylovich *et al.*, 2011; Sarova *et al.*, 2006).

### 2.1. Synthesis and crystallization

The platinum complex was synthesized by the described method (Bolotin *et al.*, 2013). Crystals of I suitable for X-ray diffraction were obtained by a slow evaporation of a nitro­methane solution of the complex at room temperature in air.

### 2.2. Refinement

Crystal data, data collection and structure refinement details are summarized in Table 1. The solvent of crystallization (MeNO_2_) was disordered over two sites about an inversion centre with equal occupancies. The NH hydrogen atoms were located from the difference Fourier map but constrained to ride on their parent atom, with *U*_iso_ = 1.5 U_eq_(parent atom). Other hydrogen atoms were positioned geometrically and constrained to ride on their parent atoms, with C—H = 0.95-0.98 Å, and *U*_iso_ = 1.2-1.5 *U*_eq_(parent atom). The highest peak is located 0.68 Å from atom Pt1 and the deepest hole is located 0.64 Å from atom Pt1.

### 3. Results and discussion

Compound I crystallizes from MeNO_2_ as hemisolvate [PtCl_2_(C_13_H_21_N_5_)].0.5MeNO_2_. Nitro­methane molecules incorporated in the crystals lattice are disordered over two sites with equal occupancies. The coordination polyhedron of platinum exhibits a typical square-planar geometry. All bond angles around the Pt^II^ center are close to 90°. The Pt–Cl distances (2.3223 (3) and 2.3279 (3) Å) are specific for the Pt^II^–Cl bonds, the Pt–N_imine_ bond length of 1.9809 (11) Å is a characteristic value in (imine)Pt^II^ species, while the Pt–N_hydrazone_ bond length (2.0309 (11) Å) is characteristic for Pt^II^–N^sp2^ complexes (Orpen *et al.*, 1989).

The C–N_imine_ and C–N_hydrazone_ bond lengths are typical C=N double bonds, equal to 1.2966 (16) Å and 1.3049 (16) Å, respectively, while the amide N–(C=N)_imine_, N–(C=N)_hydrazone_ and the C–NMe_2_ distances are close to normal single bond values [1.3826 (17), 1.3660 (17), and 1.3408 (17) Å, respectively] (Allen *et al.*, 1987). The N–N distance of 1.4402 (16) Å is specific for a normal single N^sp2^–N^sp3^ bond (Allen *et al.*, 1987).

In the molecular structure, the (1,3,5-tri­aza­penta­diene)Pt^II^ ring is slightly bent and does not conjugate with the aromatic ring. The dihedral angle between the mean plane of the aromatic ring C4/C10 and the atoms N1/C3/N3 is 73.76 (9)°. All bond angles in this metallacycle are close to 120°, except the (N=C)_imine_–N–(C=N)_hydrazone_ angle, which is equal to 127.96 (11)° and the N–Pt–N angle, which is close to 90° [88.49 (5)°]. Weak inter­molecular H-bondings between the amidoxime amide group and one of the chlorine atoms were observed in the crystal structure.

### Crystal data

**Table:**

[PtCl_2_(C_13_H_21_N_5_)]·0.5CH_3_NO_2_	*F*(000) = 2096
*M**_r_* = 543.86	*D*_x_ = 1.958 Mg m^−^^3^
Monoclinic, *C*2/*c*	Mo *K*α radiation, λ = 0.71073 Å
Hall symbol: -C 2yc	Cell parameters from 9933 reflections
*a* = 20.7561 (3) Å	θ = 3.2–36.4°
*b* = 15.1847 (3) Å	µ = 7.91 mm^−^^1^
*c* = 14.5191 (2) Å	*T* = 100 K
β = 126.255 (1)°	Block, yellow
*V* = 3690.1 (1) Å^3^	0.44 × 0.29 × 0.20 mm
*Z* = 8	

### Data collection

**Table:**

Bruker Kappa APEXII DUO CCD diffractometer	9015 independent reflections
Radiation source: fine-focus sealed tube	8276 reflections with *I* > 2σ(*I*)
Curved graphite crystal monochromator	*R*_int_ = 0.017
Detector resolution: 16 pixels mm^-1^	θ_max_ = 36.5°, θ_min_ = 2.0°
φ scans and ω scans with κ offset	*h* = −34→33
Absorption correction: numerical (*SADABS*; Sheldrick, 2008*a*)	*k* = −25→24
*T*_min_ = 0.130, *T*_max_ = 0.297	*l* = −20→24
38976 measured reflections	

### Refinement

**Table:**

Refinement on *F*^2^	Primary atom site location: structure-invariant direct methods
Least-squares matrix: full	Secondary atom site location: difference Fourier map
*R*[*F*^2^ > 2σ(*F*^2^)] = 0.015	Hydrogen site location: inferred from neighbouring sites
*wR*(*F*^2^) = 0.035	H-atom parameters constrained
*S* = 1.06	*w* = 1/[σ^2^(*F*_o_^2^) + (0.0133*P*)^2^ + 5.9064*P*] where *P* = (*F*_o_^2^ + 2*F*_c_^2^)/3
9015 reflections	(Δ/σ)_max_ = 0.002
226 parameters	Δρ_max_ = 0.78 e Å^−^^3^
2 restraints	Δρ_min_ = −1.01 e Å^−^^3^

### Special details

**Table:**

Geometry. All e.s.d.'s (except the e.s.d. in the dihedral angle between two l.s. planes) are estimated using the full covariance matrix. The cell e.s.d.'s are taken into account individually in the estimation of e.s.d.'s in distances, angles and torsion angles; correlations between e.s.d.'s in cell parameters are only used when they are defined by crystal symmetry. An approximate (isotropic) treatment of cell e.s.d.'s is used for estimating e.s.d.'s involving l.s. planes.
Refinement. Refinement of *F*^2^ against ALL reflections. The weighted *R*-factor *wR* and goodness of fit *S* are based on *F*^2^, conventional *R*-factors *R* are based on *F*, with *F* set to zero for negative *F*^2^. The threshold expression of *F*^2^ > σ(*F*^2^) is used only for calculating *R*-factors(gt) *etc*. and is not relevant to the choice of reflections for refinement. *R*-factors based on *F*^2^ are statistically about twice as large as those based on *F*, and *R*- factors based on ALL data will be even larger.

### Fractional atomic coordinates and isotropic or equivalent isotropic displacement parameters (Å^2^)

**Table:**

	*x*	*y*	*z*	*U*_iso_*/*U*_eq_	Occ. (<1)
Pt1	0.086711 (3)	0.544103 (3)	0.926821 (4)	0.01177 (1)	
Cl1	0.101415 (19)	0.39718 (2)	0.89820 (3)	0.01527 (5)	
Cl2	0.22139 (2)	0.56194 (2)	1.00525 (4)	0.02319 (7)	
N1	0.06341 (7)	0.66831 (7)	0.95151 (10)	0.01384 (18)	
N2	0.10725 (7)	0.74645 (8)	0.96392 (11)	0.0178 (2)	
N3	−0.05540 (7)	0.62871 (8)	0.92740 (10)	0.01448 (18)	
H3N	−0.0805	0.6409	0.9571	0.022*	
N4	−0.15323 (7)	0.52601 (8)	0.81191 (10)	0.01497 (19)	
N5	−0.02759 (7)	0.51555 (8)	0.84918 (10)	0.01401 (18)	
H5N	−0.0494	0.4762	0.7988	0.021*	
C1	0.11579 (9)	0.75208 (11)	0.87141 (14)	0.0222 (3)	
H1A	0.1448	0.7003	0.8734	0.033*	
H1B	0.1455	0.8055	0.8806	0.033*	
H1C	0.0628	0.7542	0.7980	0.033*	
C2	0.18337 (9)	0.75253 (11)	1.07686 (14)	0.0223 (3)	
H2A	0.1735	0.7596	1.1346	0.033*	
H2B	0.2134	0.8034	1.0793	0.033*	
H2C	0.2143	0.6987	1.0925	0.033*	
C3	0.00349 (8)	0.68722 (8)	0.95483 (11)	0.01326 (19)	
C4	−0.00996 (8)	0.77467 (9)	0.98743 (11)	0.0143 (2)	
C5	0.03328 (10)	0.80236 (10)	1.10020 (13)	0.0214 (3)	
H5A	0.0757	0.7671	1.1591	0.026*	
C6	0.01455 (10)	0.88207 (11)	1.12719 (14)	0.0241 (3)	
H6	0.0446	0.9005	1.2047	0.029*	
C7	−0.04716 (9)	0.93496 (10)	1.04312 (14)	0.0204 (3)	
C8	−0.06930 (11)	1.01952 (11)	1.07194 (18)	0.0287 (3)	
H8A	−0.1182	1.0107	1.0665	0.043*	
H8B	−0.0783	1.0656	1.0182	0.043*	
H8C	−0.0258	1.0374	1.1499	0.043*	
C9	−0.09025 (9)	0.90607 (10)	0.93054 (14)	0.0220 (3)	
H9	−0.1323	0.9416	0.8715	0.026*	
C10	−0.07310 (9)	0.82650 (10)	0.90270 (13)	0.0196 (2)	
H10	−0.1045	0.8072	0.8255	0.023*	
C11	−0.07838 (8)	0.55407 (8)	0.86030 (11)	0.01253 (19)	
C12	−0.17809 (9)	0.43894 (9)	0.76070 (13)	0.0182 (2)	
H12A	−0.2021	0.4431	0.6790	0.027*	
H12B	−0.2174	0.4151	0.7709	0.027*	
H12C	−0.1315	0.3999	0.7977	0.027*	
C13	−0.21699 (8)	0.58213 (10)	0.79291 (13)	0.0197 (2)	
H13A	−0.2321	0.5628	0.8421	0.030*	
H13B	−0.2634	0.5781	0.7127	0.030*	
H13C	−0.1984	0.6433	0.8111	0.030*	
O1	0.23109 (19)	0.25839 (19)	0.8531 (2)	0.0342 (6)	0.50
N6	0.24020 (19)	0.2509 (2)	0.9450 (3)	0.0313 (6)	0.50
C14	0.1897 (11)	0.1872 (12)	0.958 (2)	0.0371 (7)	0.50
H14A	0.1352	0.1861	0.8870	0.056*	0.50
H14B	0.1883	0.2062	1.0211	0.056*	0.50
H14C	0.2129	0.1280	0.9737	0.056*	0.50
O2	0.1983 (8)	0.1969 (8)	0.9556 (13)	0.0371 (7)	0.50

### Atomic displacement parameters (Å^2^)

**Table:**

	*U*^11^	*U*^22^	*U*^33^	*U*^12^	*U*^13^	*U*^23^
Pt1	0.01165 (2)	0.01170 (2)	0.01451 (2)	0.00007 (1)	0.00913 (2)	−0.00061 (1)
Cl1	0.01681 (12)	0.01324 (12)	0.01993 (12)	0.00183 (10)	0.01316 (11)	0.00001 (10)
Cl2	0.01519 (13)	0.02011 (15)	0.03501 (18)	−0.00179 (11)	0.01526 (14)	−0.00348 (13)
N1	0.0138 (4)	0.0127 (4)	0.0187 (5)	−0.0007 (3)	0.0116 (4)	−0.0007 (4)
N2	0.0186 (5)	0.0140 (5)	0.0267 (6)	−0.0036 (4)	0.0167 (5)	−0.0011 (4)
N3	0.0164 (4)	0.0130 (4)	0.0197 (5)	−0.0019 (4)	0.0138 (4)	−0.0030 (4)
N4	0.0124 (4)	0.0142 (5)	0.0196 (5)	−0.0008 (3)	0.0102 (4)	−0.0019 (4)
N5	0.0132 (4)	0.0136 (5)	0.0165 (4)	−0.0017 (4)	0.0095 (4)	−0.0027 (4)
C1	0.0186 (6)	0.0260 (7)	0.0252 (6)	−0.0016 (5)	0.0146 (5)	0.0058 (5)
C2	0.0220 (6)	0.0211 (6)	0.0266 (7)	−0.0084 (5)	0.0159 (6)	−0.0085 (5)
C3	0.0149 (5)	0.0120 (5)	0.0158 (5)	−0.0003 (4)	0.0106 (4)	−0.0001 (4)
C4	0.0155 (5)	0.0120 (5)	0.0192 (5)	−0.0003 (4)	0.0124 (5)	−0.0009 (4)
C5	0.0278 (7)	0.0177 (6)	0.0187 (6)	0.0067 (5)	0.0137 (5)	0.0000 (5)
C6	0.0300 (7)	0.0202 (7)	0.0243 (6)	0.0044 (5)	0.0172 (6)	−0.0040 (5)
C7	0.0229 (6)	0.0132 (5)	0.0346 (7)	0.0003 (5)	0.0222 (6)	−0.0019 (5)
C8	0.0349 (8)	0.0158 (6)	0.0492 (10)	0.0023 (6)	0.0326 (8)	−0.0035 (6)
C9	0.0180 (6)	0.0179 (6)	0.0303 (7)	0.0045 (5)	0.0144 (6)	0.0018 (5)
C10	0.0165 (5)	0.0180 (6)	0.0226 (6)	0.0025 (5)	0.0107 (5)	−0.0013 (5)
C11	0.0133 (5)	0.0117 (5)	0.0146 (5)	0.0000 (4)	0.0094 (4)	0.0005 (4)
C12	0.0175 (5)	0.0166 (6)	0.0221 (6)	−0.0038 (4)	0.0126 (5)	−0.0042 (5)
C13	0.0138 (5)	0.0198 (6)	0.0246 (6)	0.0040 (4)	0.0107 (5)	0.0023 (5)
O1	0.0462 (16)	0.0301 (13)	0.0322 (13)	0.0129 (12)	0.0263 (13)	0.0062 (10)
N6	0.0300 (14)	0.0254 (14)	0.0396 (16)	0.0101 (11)	0.0211 (13)	0.0053 (12)
C14	0.032 (3)	0.028 (3)	0.0494 (12)	−0.0033 (15)	0.0232 (15)	0.0019 (18)
O2	0.032 (3)	0.028 (3)	0.0494 (12)	−0.0033 (15)	0.0232 (15)	0.0019 (18)

### Geometric parameters (Å, º)

**Table:**

Pt1—N5	1.9809 (11)	C4—C10	1.3941 (19)
Pt1—N1	2.0309 (11)	C5—C6	1.396 (2)
Pt1—Cl1	2.3223 (3)	C5—H5A	0.9500
Pt1—Cl2	2.3279 (3)	C6—C7	1.388 (2)
N1—C3	1.3049 (16)	C6—H6	0.9500
N1—N2	1.4402 (16)	C7—C9	1.391 (2)
N2—C1	1.4576 (19)	C7—C8	1.504 (2)
N2—C2	1.459 (2)	C8—H8A	0.9800
N3—C3	1.3660 (17)	C8—H8B	0.9800
N3—C11	1.3826 (17)	C8—H8C	0.9800
N3—H3N	0.8702	C9—C10	1.386 (2)
N4—C11	1.3408 (17)	C9—H9	0.9500
N4—C12	1.4537 (18)	C10—H10	0.9500
N4—C13	1.4567 (18)	C12—H12A	0.9800
N5—C11	1.2966 (16)	C12—H12B	0.9800
N5—H5N	0.8399	C12—H12C	0.9800
C1—H1A	0.9800	C13—H13A	0.9800
C1—H1B	0.9800	C13—H13B	0.9800
C1—H1C	0.9800	C13—H13C	0.9800
C2—H2A	0.9800	O1—N6	1.237 (3)
C2—H2B	0.9800	N6—C14	1.518 (8)
C2—H2C	0.9800	C14—H14A	0.9800
C3—C4	1.4904 (18)	C14—H14B	0.9800
C4—C5	1.3875 (19)	C14—H14C	0.9800
			
N5—Pt1—N1	88.49 (5)	C10—C4—C3	118.53 (12)
N5—Pt1—Cl1	85.86 (3)	C4—C5—C6	120.05 (14)
N1—Pt1—Cl1	173.69 (3)	C4—C5—H5A	120.0
N5—Pt1—Cl2	172.48 (3)	C6—C5—H5A	120.0
N1—Pt1—Cl2	98.51 (3)	C7—C6—C5	121.33 (14)
Cl1—Pt1—Cl2	87.274 (12)	C7—C6—H6	119.3
C3—N1—N2	111.07 (11)	C5—C6—H6	119.3
C3—N1—Pt1	122.86 (9)	C6—C7—C9	117.95 (13)
N2—N1—Pt1	126.04 (8)	C6—C7—C8	121.61 (15)
N1—N2—C1	110.19 (11)	C9—C7—C8	120.41 (15)
N1—N2—C2	112.14 (11)	C7—C8—H8A	109.5
C1—N2—C2	113.01 (11)	C7—C8—H8B	109.5
C3—N3—C11	127.96 (11)	H8A—C8—H8B	109.5
C3—N3—H3N	114.3	C7—C8—H8C	109.5
C11—N3—H3N	117.7	H8A—C8—H8C	109.5
C11—N4—C12	120.52 (11)	H8B—C8—H8C	109.5
C11—N4—C13	123.69 (12)	C10—C9—C7	121.34 (14)
C12—N4—C13	115.29 (11)	C10—C9—H9	119.3
C11—N5—Pt1	128.23 (9)	C7—C9—H9	119.3
C11—N5—H5N	111.7	C9—C10—C4	120.30 (14)
Pt1—N5—H5N	119.9	C9—C10—H10	119.9
N2—C1—H1A	109.5	C4—C10—H10	119.9
N2—C1—H1B	109.5	N5—C11—N4	124.54 (12)
H1A—C1—H1B	109.5	N5—C11—N3	119.36 (12)
N2—C1—H1C	109.5	N4—C11—N3	116.09 (11)
H1A—C1—H1C	109.5	N4—C12—H12A	109.5
H1B—C1—H1C	109.5	N4—C12—H12B	109.5
N2—C2—H2A	109.5	H12A—C12—H12B	109.5
N2—C2—H2B	109.5	N4—C12—H12C	109.5
H2A—C2—H2B	109.5	H12A—C12—H12C	109.5
N2—C2—H2C	109.5	H12B—C12—H12C	109.5
H2A—C2—H2C	109.5	N4—C13—H13A	109.5
H2B—C2—H2C	109.5	N4—C13—H13B	109.5
N1—C3—N3	123.64 (12)	H13A—C13—H13B	109.5
N1—C3—C4	124.71 (12)	N4—C13—H13C	109.5
N3—C3—C4	111.65 (11)	H13A—C13—H13C	109.5
C5—C4—C10	118.99 (13)	H13B—C13—H13C	109.5
C5—C4—C3	122.20 (12)	O1—N6—C14	121.0 (10)
			
N5—Pt1—N1—C3	24.61 (11)	N3—C3—C4—C10	−70.06 (15)
Cl2—Pt1—N1—C3	−158.15 (10)	C10—C4—C5—C6	−1.5 (2)
N5—Pt1—N1—N2	−153.35 (11)	C3—C4—C5—C6	−175.32 (14)
Cl2—Pt1—N1—N2	23.89 (11)	C4—C5—C6—C7	0.2 (3)
C3—N1—N2—C1	−127.66 (12)	C5—C6—C7—C9	0.2 (2)
Pt1—N1—N2—C1	50.50 (15)	C5—C6—C7—C8	178.10 (16)
C3—N1—N2—C2	105.52 (13)	C6—C7—C9—C10	0.8 (2)
Pt1—N1—N2—C2	−76.31 (14)	C8—C7—C9—C10	−177.14 (15)
N1—Pt1—N5—C11	−23.40 (12)	C7—C9—C10—C4	−2.2 (2)
Cl1—Pt1—N5—C11	153.79 (12)	C5—C4—C10—C9	2.5 (2)
N2—N1—C3—N3	169.27 (12)	C3—C4—C10—C9	176.52 (13)
Pt1—N1—C3—N3	−8.97 (18)	Pt1—N5—C11—N4	−173.99 (10)
N2—N1—C3—C4	−10.04 (18)	Pt1—N5—C11—N3	5.04 (18)
Pt1—N1—C3—C4	171.73 (9)	C12—N4—C11—N5	12.4 (2)
C11—N3—C3—N1	−22.1 (2)	C13—N4—C11—N5	−159.13 (13)
C11—N3—C3—C4	157.24 (12)	C12—N4—C11—N3	−166.66 (12)
N1—C3—C4—C5	−76.83 (18)	C13—N4—C11—N3	21.81 (19)
N3—C3—C4—C5	103.79 (15)	C3—N3—C11—N5	24.5 (2)
N1—C3—C4—C10	109.32 (16)	C3—N3—C11—N4	−156.34 (13)

### Hydrogen-bond geometry (Å, º)

**Table:**

*D*—H···*A*	*D*—H	H···*A*	*D*···*A*	*D*—H···*A*
N3—H3*N*···Cl1^i^	0.87	2.45	3.2154 (11)	147
N5—H5*N*···Cl1^ii^	0.84	2.67	3.4598 (12)	157

Symmetry codes: (i) −*x*, −*y*+1, −*z*+2; (ii) −*x*, *y*, −*z*+3/2.
